# Implementation of proteomic biomarkers: making it work

**DOI:** 10.1111/j.1365-2362.2012.02674.x

**Published:** 2012-09

**Authors:** Harald Mischak, John PA Ioannidis, Angel Argiles, Teresa K Attwood, Erik Bongcam-Rudloff, Mark Broenstrup, Aristidis Charonis, George P Chrousos, Christian Delles, Anna Dominiczak, Tomasz Dylag, Jochen Ehrich, Jesus Egido, Peter Findeisen, Joachim Jankowski, Robert W Johnson, Bruce A Julien, Tim Lankisch, Hing Y Leung, David Maahs, Fulvio Magni, Michael P Manns, Efthymios Manolis, Gert Mayer, Gerjan Navis, Jan Novak, Alberto Ortiz, Frederik Persson, Karlheinz Peter, Hans H Riese, Peter Rossing, Naveed Sattar, Goce Spasovski, Visith Thongboonkerd, Raymond Vanholder, Joost P Schanstra, Antonia Vlahou

**Affiliations:** 1 BHF Glasgow Cardiovascular Research Centre, Institute of Cardiovascular and Medical Sciences, College of Medical, Veterinary and Life Sciences, University of GlasgowGlasgow, UK; 2 Mosaiques diagnosticsHannover, Germany; 3 Clinical and Molecular Epidemiology Unit, Department of Hygiene and Epidemiology, University of Ioannina School of MedicineIoannina, Greece; 4 Stanford Prevention Research Center, Department of Medicine, Stanford University School of MedicineStanford, CA, USA; 5 Department of Health Research and Policy, Stanford University School of MedicineStanford, CA, USA; 6 Department of Statistics, Stanford University School of Humanities and SciencesStanford, CA, USA; 7 RD NéphrologieMontpellier Cedex, France; 8 Faculty of Life Sciences and School of Computer Science, University of ManchesterManchester, UK; 9 Department of Immunology, Genetics and Pathology, Uppsala UniversityUppsala, Sweden; 10 Department of Animal Breeding and Genetics, Swedish University of Agricultural SciencesUppsala, Sweden; 11 Sanofi-Aventis Deutschland GmbH D-65926 Frankfurt am MainGermany; 12 Divisions of Histology and Biotechnology, Biomedical Research Foundation, Academy of AthensAthens, Greece; 13 First Department of Pediatrics, Athens University Medical School, Aghia Sophia Children's HospitalAthens, Greece; 14 European Commission, Directorate-General for Research and Innovation, Unit F5: Personalised Medicine21 rue du Champ de Mars, Brussels, Belgium; 15 Hannover Medical School, Department of PediatricsHannover, Germany; 16 IIS-Fundación Jiménez Díaz, Autonoma UniversityMadrid, Spain; 17 C Institute of Clinical Chemistry, Universitätsklinikum MannheimMannheim, Germany; 18 Charité (CBF), Medizinische Klinik IVBerlin, Germany; 19 Abbott LaboratoriesAbbott Park, IL, USA; 20 Department of Medicine, University of Alabama at BirminghamBirmingham, AL, USA; 21 Department of Gastroenterology, Hepatology and Endocrinology, Hannover Medical SchoolHannover, Germany; 22 The Beatson Institute for Cancer ResearchGarscube Estate, Glasgow, UK; 23 Barbara Davis Center for Childhood Diabetes, University of Colorado DenverAurora, CO, USA; 24 Department of Experimental Medicine, University of Milano-BicoccaMonza, Italy; 25 European Medicines AgencyLondon, UK; 26 Department of Internal Medicine IV, Medical University of InnsbruckInnsbruck, Austria; 27 Division of Nephrology, Department of Internal Medicine, University Medical Centre Groningen, University of GroningenGroningen, The Netherlands; 28 Department of Microbiology, University of Alabama at BirminghamBirmingham, AL, USA; 29 Steno Diabetes CenterGentofte, Denmark; 30 Baker Heart Research InstituteMelbourne, Victoria, Australia; 31 European Projects Office, Institute of Health Carlos IIIMadrid, Spain; 32 Department of Nephrology, Medical Faculty, University of SkopjeSkopje, Former Yugoslav Republic of Macedonia; 33 Faculty of Medicine at Siriraj Hospital, Mahidol UniversityBangkok, Thailand; 34 Department of Nephrology, Ghent University HospitalGhent, Belgium; 35 Institut National de la Santé et de la Recherche Médicale (INSERM), U1048, Institut of Cardiovascular and Metabolic Disease and Université Toulouse III Paul-SabatierToulouse, France; 36 Division of Biotechnology, Biomedical Research Foundation, Academy of Athens11527 Athens, Greece

**Keywords:** Biomarker, biomarker implementation, clinical proteomics, clinical studies, expert panel, proteomics

## Abstract

While large numbers of proteomic biomarkers have been described, they are generally not implemented in medical practice. We have investigated the reasons for this shortcoming, focusing on hurdles downstream of biomarker verification, and describe major obstacles and possible solutions to ease valid biomarker implementation. Some of the problems lie in suboptimal biomarker discovery and validation, especially lack of validated platforms with well-described performance characteristics to support biomarker qualification. These issues have been acknowledged and are being addressed, raising the hope that valid biomarkers may start accumulating in the foreseeable future. However, successful biomarker discovery and qualification alone does not suffice for successful implementation. Additional challenges include, among others, limited access to appropriate specimens and insufficient funding, the need to validate new biomarker utility in interventional trials, and large communication gaps between the parties involved in implementation. To address this problem, we propose an implementation roadmap. The implementation effort needs to involve a wide variety of stakeholders (clinicians, statisticians, health economists, and representatives of patient groups, health insurance, pharmaceutical companies, biobanks, and regulatory agencies). Knowledgeable panels with adequate representation of all these stakeholders may facilitate biomarker evaluation and guide implementation for the specific context of use. This approach may avoid unwarranted delays or failure to implement potentially useful biomarkers, and may expedite meaningful contributions of the biomarker community to healthcare.

## Introduction

Clinical proteomics is defined as proteome analysis intended to improve the medical practice, for example, in relation to diagnosis, prevention, prognosis or therapy. Its success should be judged from the conferred clinical impact after implementation of its findings in everyday practice. The last decade has been marked by significant technological advancements in proteomics, especially with regard to mass spectrometry and bioinformatic solutions for data analysis. Over 4000 manuscripts including the words ‘clinical’ and ‘proteomics’ were indexed in MEDLINE in the last decade. Multiple proteomic biomarkers have been described for a variety of diseases, and several biomarkers have shown added value over current disease-management approaches, based on validation studies (e.g. in chronic kidney disease [1–3], reviewed in [4,5]). Nevertheless, implementation of the results in medical practice appears to be scarce [6]. Despite the promising findings, the impact of clinical proteomics (and biomarkers in general) on clinical decision-making, patient management and welfare appears insufficient.

Much of the problem may still lie in suboptimal discovery and validation processes for proteomic and other highly touted biomarkers. Analytical validation must be done prior to even starting a study, and the performance characteristics of the platform must be known [7–11]. Empirical evidence has shown that even in the best-studied and most studied biomarkers from diverse fields beyond proteomics, initial expectations may be inflated, and true effects may be much smaller than originally believed [12,13]. As others have pointed out, a plethora of factors can before, during and after sample analysis complicate biomarker discovery and validation and lead to false discoveries [14]. Nevertheless, it is to be expected that, as these factors are more clearly recognised, discovery and validation processes might be improved to a point where they are no longer the main bottleneck to progress. Enhanced attention is already given to clinical proteomics workflows, with special emphasis on experimental design of biomarker discovery, standardisation of procedures, data analysis and interpretation of results [11,15–17]. Mandatory requirements for contributors have, to some degree, been adopted by scientific journals (e.g. http://www.mcponline.org/site/cpmeeting/cguidelines.pdf). Technological bottlenecks associated with the transformation of discoveries into potential clinical assay are being identified and addressed [18–21]. Hopefully, if these efforts and insights become systematically exploited and implemented, valid biomarkers may start accumulating in the foreseeable future, perhaps even at a rapid pace. However, even then, successful biomarker discovery and qualification alone does not suffice for successful clinical implementation. The objective of this article is to highlight the critical hurdles downstream of biomarker discovery and verification, and to suggest potential ways to overcome them.

## Challenges in transforming biomarker discoveries into clinical application

Frequently, the discovery of biomarkers is considered a successful endpoint of clinical proteomics. Discovery and publication is a prerequisite for biomarker development, but it must be transformed into the ultimate goal of this particular translational proteomics research: clinical application. While highly ranked publications can have a prompt and major personal impact on the involved scientists (e.g. increased funding and advancement of academic career), the actual implementation requires substantially more time and is associated with diverse, unforeseeable challenges, which can bring the process to a standstill. Many scientists appear unwilling to venture down this tortuous and uncertain path.

In addition, different categories of biomarkers exist, depending on their intended use, as defined, for example, by Khleif *et al.* [22]: A biomarker is a characteristic that is objectively measured and evaluated as an indicator of normal biological processes, pathogenic processes or pharmacologic responses to a therapeutic intervention. At least, four different categories of biomarkers should be differentiated: (i) diagnostic biomarkers (early detection biomarkers, disease classification); (ii) predictive biomarkers (predict patients likely to respond to a specific agent, predict patients likely to have an adverse event to a specific agent); (iii) metabolism biomarkers (dose defining); and (iv) outcome biomarkers (forecast response, progression or recurrence).

Implementation requires demonstration of clinical validity and utility, and benefit for the patient [23,24]. This process is demanding in time, as well as clinical, scientific and financial resources, and generally requires large studies. These include the assessment of performance on introduction of the novel biomarker over and above routinely available information; randomised trials to test improvement in clinical outcomes by using the biomarker; and late implementation and dissemination studies to show that the biomarker was successfully applied in everyday practice, with improved outcomes in large populations and a concomitant decrease in the cost of care – or, at least, without a substantial increase [25]. Such implementation testing is likely to take years, often exceeding a decade, and continues even after the biomarker has been applied and used widely in the community.

Biomarkers are actively sought for the majority of diseases associated with major societal and economic burden in developed countries (e.g. dementia, renal and cardiovascular disease, and most malignancies). However, it may generally take many years to clearly demonstrate the value of incorporating these biomarkers in management decisions in randomised trials that are evaluated based on hard endpoints. Possible solutions with shorter time horizons include the use of surrogate endpoints and/or the analysis of biomarkers in already available collections of samples with known outcomes. Surrogate endpoints may sometimes be misleading [26]. Therefore, there is debate about whether clinical implementation should be based only on results from studies that assess hard endpoints, or whether lower-level evidence that can be assessed faster may suffice. An expedited approval and implementation process based on surrogate and/or retrospective evidence may carry the risk of introducing expensive, useless or even harmful tests [27]. On the other hand, withholding an apparently beneficial test may deny a benefit for patients. As a consequence, a decision needs to be made at an early point in time whether evidence based on surrogates will be acceptable: for example, whether there is the potential for major health gains by introducing a biomarker with only modest evidence to support its use, or whether a hard endpoint must be assessed before implementation.

Evaluation of biomarkers based on analysis of previously collected samples with known outcome can be useful for the preliminary assessment of predictive or diagnostic efficacy, and the efficient reclassification of participants into informative risk categories with different implications for preventive or therapeutic intervention. However, such studies may involve a selection bias and do not guarantee that the use of biomarkers would improve the clinical outcome.

Another major impediment to implementation is that scientists are generally not well informed about the required steps from initial discovery to translation into a clinically useful assay. In fact, a clear road map towards implementation does not exist and guidance is scarce. Furthermore, regulatory requirements, if existing and applicable, are generally unknown to most researchers. This uncertainty and lack of adequate knowledge, in combination with the aforementioned need for substantial efforts and funding to demonstrate clinical validity, utility and added value of biomarkers over current clinical standards through, in principle, large trials, generally bring the further development of discovery findings to a standstill.

Performance of biomarkers superior to that for current standards does not automatically result in actual clinical implementation for several additional reasons: (i) biomarkers, even if facilitating substantial improvements in patient assessment, may initially fall short in influencing patient management, owing to the lack of appropriate interventions; (ii) patients may not wish to know about their risk of disease, especially if there is no proven intervention available; (iii) beyond the value at the clinical level, a biomarker must prove its cost-effectiveness [17], and this may differ by country, healthcare system and study approach applied by health economists; and (iv) physicians may resist changing the *status quo* in daily clinical practice, especially if this change is associated with personal financial consequences (procedures profitable for the physicians are not likely to be replaced).

Substantial uncertainty exists about the road towards implementation of biomarkers. There are multiple, interrelated steps where a plethora of different parties are involved, including researchers, clinicians, healthcare providers, funding and regulatory agencies, legislative, educative, and health insurance bodies, industry and patient groups. Each of these groups views the implementation from a different and unique angle, and cross-communication is often challenging. This results in fragmentation and severe gaps in the flow of information that may impede the clinical translation of research advancements.

## A real-life example

The following typical scenario exemplifies how various challenges may create interactive hurdles for implementation of properly qualified biomarkers. Early detection of diabetic nephropathy, followed by appropriate therapy, is expected to prevent progression to advanced renal disease [28,29], improve quality of life and life expectancy of patients, and reduce the societal economic burden. Considering that biomarkers have been discovered and that associations of specific proteins or protein patterns have been validated in blinded studies (e.g. [2]), the next step would be to further validate these markers in prospective clinical trials. Of paramount importance are the definition of the endpoint(s) (in this case, detection of progression of chronic kidney disease – for example, end-stage renal disease with need for renal replacement therapy) and proof of an at least incremental superiority to existing diagnostic standards (e.g. assessment of albuminuria). The ideal study for such a chronic, slowly progressive disease with a substantial proportion of diabetic patients not affected requires the prospective collection of samples over many years in a large population for the accumulation of sufficient data to evaluate a hard endpoint. This long time-frame is a major setback for everybody. To exemplify the challenge, several of the major trials in the area are listed in Table 1. In most trials, only surrogate endpoints based on albuminuria were assessed, and, for several of the trials, hard endpoints for chronic kidney disease (doubling of serum creatinine concentration or end-stage renal disease) were not reported. Unfortunately, collection of follow-up data to assess hard endpoints at a later point in time was generally not foreseen. Apparent benefit of intervention based on a surrogate parameter (reduction of albuminuria) has been demonstrated in currently manifested stages of disease [28,29], but one needs to extrapolate whether a benefit would apply also to earlier stages. Patients with diabetes may be reluctant to be informed about early signs of kidney disease (e.g. years before recurrent microalbuminuria), given that many current therapeutic options are not proven to be effective at that stage, while potentially more effective intervention measures are still under development. We are hence faced with a fundamental question: Shall we implement such biomarkers now and employ current intervention strategies based on the assumption they will bring a significant benefit at early stages of disease, or shall we, prior to implementation, investigate whether the intervention strategies bring a significant benefit, either based on surrogate endpoints (e.g. albuminuria) or on hard endpoints? Thus, while prevention of diabetic nephropathy (or any other disease) is a worthy goal, the implementation path is far less clear.

**Table 1:** Duration, demographic parameters and outcome of major trials testing early intervention in diabetic nephropathy

	DIRECT I	DIRECT II	HOPE	BENEDICT	ADVANCE	ROADMAP
Treated (*N*)	1662	951	1808	601	5569	2232
Placebo (*N*)	1664	954	1769	603	5571	2215
Age (years)	31	57	65	62	66	57·7
Diabetes type	Type I	Type II	Type II	Type II	Type II	Type II
Diabetes duration (years)	9·1	8·8	11	8	8	6
BP (Systol/diastol)	117/73	133/78	142/80	151/88	145/81	136/81
Active treatment	Candesartan	Candesartan	Ramipril	Trandolapril	Perindopril/Indapamide	Olmesartan
Duration of treatment (years)	4·7	4·7	4·5	3·6	4·3	3·2
Incidence of new microalbuminuria (%)	5·0/5·0	12·0/13·0	33/38	5·8/11	19·6/23·6	8·2/9·8
Doubling of Screa (*N*)	NA	NA	NA	NA	55/45	23/23
ESRD (*N*)	NA	NA	10/8	NA	25/21	0/0
Death (*N*)	14/13	37/35	196/248	12	408/471	26/15
Rate of death (%/year)	0·17	0·80	2·76	0·28	1·83	0·29
Rate of onset ESRD (%/year)	NA	NA	0·11	NA	0·10	0·00
Rate of doubling of Screa (%/year)	NA	NA	NA	NA	0·21	0·43

BP, blood pressure; Screa, serum creatinine; ESRD, end stage renal disease; NA, not accessible.

Data reported were extracted from DIRECT I [41], DIRECT II [42,43], HOPE [44], BENEDICT [28], ADVANCE [45] and ROADMAP [29]. While most trials demonstrated a positive effect of intervention when assessing a surrogate parameter, albuminuria, a benefit based on hard endpoints was generally not demonstrated and was frequently not even assessed. The events are shown as number of events or percentage, as appropriate, in the active treatment/control arm.

Given these considerations, one may reflect that perhaps research in these specific areas should not be even initiated, because it will not result in any tangible impact on the current situation; or that the current situation should be altered in a way that positive results from research have a realistic chance to be implemented to improve the current clinical status. The latter option is certainly preferable, but the goals need to be rigorously defined, and critical issues must be clearly identified to advance.

## An agenda to facilitate implementation of valid biomarkers

The implementation problem is gaining increasing recognition, and actions towards improving the situation have been initiated: the substantial financial need to support biomarker validation and qualification studies has been acknowledged by funding agencies – prominent examples are the recent EU FP7 calls for proposals for collaborative projects (http://ec.europa.eu/research/participants/portal/page/fp7_calls [30]). Additional examples include the Joint Programming Initiative in Neurodegenerative Diseases, which is funding the first pilot call for research projects in ‘Optimisation of biomarkers and harmonisation of their use between clinical centres’; and the new ERA-Net TRANSCAN, which has proposed the topic ‘Validation of biomarkers for personalised cancer medicine’. In the aforementioned example of diabetic nephropathy, a clinical trial (PRIORITY; FP7 2012–2016) is being launched, exploring the potential benefit of intervention on early diabetic kidney injury, based on a panel of urinary protein biomarkers [31]; and the ‘Early Prevention of Diabetes Complications in Europe’ (e-PREDICE) was also funded to investigate changes in biomarkers for microvascular damage, endothelial function, oxidation and inflammation, conferred as a result of different drug treatments designed for the early prevention of diabetic complications. Similar efforts are also underway in many other fields and in other countries, for example, in the USA, the Early Detection Research Network of the National Cancer Institute (http://edrn.nci.nih.gov) [32]. These are certainly major advances, shifting the emphasis from biomarker discovery to clinical application. Nevertheless, the implementation process, as a whole, still appears to be substantially under-funded.

**Figure 1:**
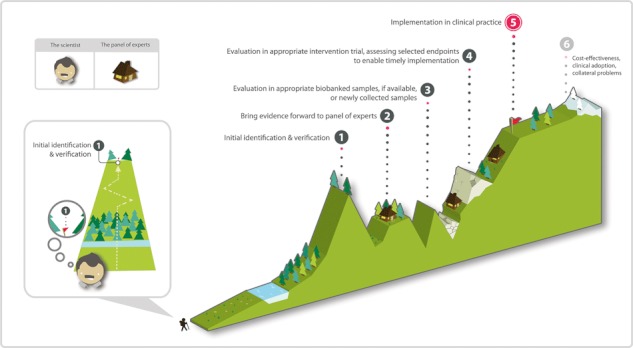
Implementation of novel biomarkers represents a substantially harder challenge than initially thought by scientists. As shown on the left-hand side of the cartoon, the current belief is that the major efforts are required during initial identification and verification of proteomics biomarkers. Implementation in the clinic is conceived as being simply a matter of continuing uphill, the ‘last few steps’ to the red flag. However, we argue that this is not true. While initial identification, verification and establishment of an appropriate analytical platform are without doubt major steps, even more substantial efforts are required on the road to actual implementation, which evidently is much longer than anticipated, and full of risks and additional obstacles. Among the major challenges are access to specific knowledge, sufficient funding, access to appropriate specimens, demonstration of reproducibility and performance of interventional trials. We propose support by a multidisciplinary panel immediately after initial verification, to accompany scientists on this road to implementation and to help avoid potentially useful biomarkers failing to reach the clinic. Once implementation in the clinic has been accomplished, the process does not stop, as cost-effectiveness, clinical adoption and collateral problems still must be monitored.

Aiming to facilitate successful implementation of research findings in medical practice, the European Medical Research Council (EMRC) recently published the ESF/EMRC ‘forward look’ (http://www.esf.org/emrc; May 2011), describing the different hurdles in the process. To spear-head an implementation road map and identify the special turns it must take in the case of proteomics findings, the European Kidney and Urine proteomics COST Action, during its regular meeting (Madrid 2011), organised a session on clinical implementation of research findings pertinent to kidney diseases. In this meeting, researchers, clinicians and representatives from biobanks, industry, funding and regulatory agencies were invited to present their views of the implementation process. Jointly, these initiatives showed that biomarker research should adhere to a much more organised format, taking into consideration the needs and perspectives of the whole spectrum of involved parties: from scientists in the discovery laboratory to end-users (patients, physicians), including regulatory bodies. We suggest the following steps to facilitate implementation of clinical proteomics findings (Fig. 1):

- Perform initial discovery and validation for the specific context of use. If positive;
- Approach a suitable multidisciplinary panel (described in detail below) to evaluate evidence, and if positive, to provide guidance for further study design;
- Apply for funding and, in parallel, request samples from biobanks, when available, or initiate new sample collection (considering the panel’s recommendations);
- Perform biomarker evaluation;
- Approach the panel for evaluation of the additional data and, if positive, for guidance for clinical study design;
- Apply for funding and perform intervention study to evaluate expected benefit. Preferably, hard endpoints should be assessed, if they can be reached in a reasonable period of time. If this is not possible, and the biomarkers are considered to have potentially life-saving clinical potential, validated surrogate endpoints may be employed, mandating additional follow-up to assess the hard endpoints;
- Approach the panel for evaluation of the evidence from the intervention study. If positive;
- Implement in clinical practice – perhaps on a limited, conditional basis until information on hard endpoints is considered robust enough;
- Apply feedback mechanisms to evaluate cost-effectiveness, clinical adoption, problems in routine application, unanticipated collateral problems.

Key issues to settle in this process include the following:

- The definition of clinical needs and desired context of use for new biomarkers should be specifically emphasised [16]. Different approaches to establish research priorities may be considered: for example, expert committees, peer review, research-gap generation or value of information modelling. A key question is who should be involved in the process and how? Some countries have established organisations to define and implement clinical priorities in a multidisciplinary and multistakeholder process, such as the Centre for Biomedical Network Research (CIBER) in Spain and the organisation INVOLVE of the UK Department of Health. Whether this is the optimal way to proceed remains to be seen. Generation of Web-based platforms allowing description of research priorities per disease and stakeholder (patient groups, health insurance companies, clinical societies, law-making bodies, etc.) appears to be a good starting-point to record existing views and to allow a more informed initiation of biomarker discovery efforts.
- A knowledgeable independent body or panel is required to evaluate the results of initial biomarker verification and qualification efforts as well as the respective claims and clinical utility in a transparent and unbiased manner. Currently, claims made for biomarkers are usually made by the scientists involved in the original studies, fostering unwarranted optimism and allegiance bias. The European Medicines Agency (EMA) and the US Food and Drug Administration (FDA) aim to provide evidence-based advice on biomarker qualification. One example is the voluntary biomarker qualification programme [33]. Biomarker data evaluation and guidance can be enhanced by the active involvement of a multidisciplinary panel (clinicians, clinical chemists, statisticians, health economists, representatives from patient groups, health insurance, pharmaceutical and biotechnology companies, (publicly funded) biobanks and regulatory agencies) that would evaluate existing data and provide specific recommendations for a road map towards clinical implementation of the biomarker for the specific context of use (including use of biobanked samples, surrogate and hard endpoints etc.; Fig. 1). Demonstration of reproducibility for markers that move towards clinical experimentation is vital; hence, evaluation should include repeatability checks by experienced statisticians/bioinformaticians using the raw data.
- Availability of funding for biomarker implementation studies. As an example, a certain part of the EU FP7 funding for collaborative projects under the Health theme is directed to clinical research projects, including investigator-driven clinical trials, to bring basic health research closer to the clinic (the 2011 call details can be found at http://ec.europa.eu/research/participants/portal/page/cooperation?callIdentifier=FP7-HEALTH-2012-INNOVATION-1). Positive evaluation of biomarker data by the panel (II) could be regarded as a positive recommendation also for funding purposes. Such implementation-focused projects should involve representatives of all parties mentioned above, as partners or members of an advisory board, to ensure timely implementation.
- Increased accessibility to biobanks. Availability of appropriate and clinically well-documented samples is frequently a major bottleneck for biomarker studies, even if all other aforementioned gaps are filled. Biobanks can largely expedite the assessment of predictive or diagnostic ability of biomarkers. Use of samples from biobanks could rapidly provide answers regarding biomarker validity and guide (or spare) further qualification studies through prospective trials [34–38]. The EU-funded Biobanking and Biomolecular Resources Research Infrastructure (BBMRI) project (http://www.bbmri.eu/) aims to build a coordinated, large-scale pan-European biobanking infrastructure, initially based on existing sample collections, resources, technologies and expertise [39]; it is intended to work as an interconnected node structure including biobanks, biomolecular resources, harmonised standards, databases and bioinformatics, as well as a platform for assessing ethical, legal and societal issues. This approach is promising; however, it has not yet been fully realised, and substantial difficulties in accessing existing biobanks are encountered. In addition, harmonisation of quality control tools and sample collection protocols, both significantly influence the results obtained, has not been achieved yet, but is mandatory. These issues not only affect *in vitro* diagnostic tests [40] but also have a major impact on multiparametric ‘-omics’ approaches and can override disease-related patterns or even abolish meaningful data interpretation.

Improvement efforts should be focused on transparency, creation of clear rules for sample accessibility and procedures for requests, including clear timelines; comprehensive listing of samples retrieved for all projects (outlining their type and the respective research project, so as to avoid duplication of efforts); streamlining of consent processes; and generation of the appropriate legislation that allows use of the stored samples. Stored samples should be associated with consent for the sample to be used in any scientific investigation. If analysing the sample is dependent on obtaining a new consent from the donor (who may well be deceased) individually for every experiment, then the value of such a biobank is minute, as its content can generally not be used because of this legal restriction.

## Conclusion

Implementation of biomarkers is a complex process that would significantly benefit from the establishment of a general road map. While we have focused on proteomic biomarkers in this article, the considerations and suggestions extend well beyond the boundaries of the proteomics community and are relevant to all new biomarker technologies.

Potential consequences of implementation should be contemplated from the very beginning of the developmental process, even at the stage of biomarker discovery. We also suggest potential means to achieve these aims. Final success, the implementation of the biomarker to benefit patients, cannot be guaranteed upfront, even if the biomarker proves to be valuable, because there is a continuous evolution of the landscape, with all of its political, societal and clinical implications. The -omics revolution, and development of personalised medicine approaches, will add greater complexity and new challenges to the implementation process in the coming years. However, if mechanisms for efficient communication and guidance on a road map towards clinical implementation are apparent and disseminated, we will be much better equipped to react more promptly and make the required adjustments. The biomarker community will be better positioned overall to make more meaningful contributions to clinical care, which after all should be the principal goal of all parties involved.

## Address

BHF Glasgow Cardiovascular Research Centre, Institute of Cardiovascular and Medical Sciences, College of Medical, Veterinary and Life Sciences, University of Glasgow, Glasgow G12 8TA, UK (H. Mischak, C. Delles, A. Dominiczak, N. Sattar); Mosaiques diagnostics, 30625 Hannover, Germany (H. Mischak); Clinical and Molecular Epidemiology Unit, Department of Hygiene and Epidemiology, University of Ioannina School of Medicine, 45110 Ioannina, Greece (J. P. A. Ioannidis); Stanford Prevention Research Center, Department of Medicine, Stanford University School of Medicine, Stanford, CA 94305, USA (J. P. A. Ioannidis); Department of Health Research and Policy, Stanford University School of Medicine, Stanford, CA 94305, USA (J. P. A. Ioannidis); Department of Statistics, Stanford University School of Humanities and Sciences, Stanford, CA 94305, USA (J. P. A. Ioannidis); RD Néphrologie, 34090 Montpellier Cedex, France (A. Argiles); Faculty of Life Sciences and School of Computer Science, University of Manchester, Manchester M13 9PL, UK (T. K. Attwood); Department of Immunology, Genetics and Pathology, Uppsala University, SE-751 24 Uppsala, Sweden (E. Bongcam-Rudloff); Department of Animal Breeding and Genetics, Swedish University of Agricultural Sciences, SE-751 24 Uppsala, Sweden (E. Bongcam-Rudloff); Sanofi-Aventis Deutschland GmbH D-65926 Frankfurt am Main, Germany (M. Broenstrup); Divisions of Histology and Biotechnology, Biomedical Research Foundation, Academy of Athens, 11527 Athens, Greece (A. Charonis); First Department of Pediatrics, Athens University Medical School, Aghia Sophia Children’s Hospital, 11527 Athens, Greece (G. P Chrousos); European Commission, Directorate-General for Research and Innovation, Unit F5: Personalised Medicine, 21 rue du Champ de Mars, Brussels, Belgium (T. Dylag); Department of Pediatrics, Hannover Medical School, 30625 Hannover, Germany (J. Ehrich); IIS-Fundación Jiménez Díaz, Autonoma University, Madrid, Spain (J. Egido, A. Ortiz); C Institute of Clinical Chemistry, Universitätsklinikum Mannheim, 68167 Mannheim, Germany (P. Findeisen); Charité (CBF), Medizinische Klinik IV, 12200 Berlin, Germany (J. Jankowski); Abbott Laboratories, Abbott Park, IL 60064, USA (R. W. Johnson); Department of Medicine, University of Alabama at Birmingham, Birmingham, AL 35294, USA (B. A. Julien); Department of Gastroenterology, Hepatology and Endocrinology, Hannover Medical School, 30625 Hannover, Germany (T. Lankisch, M. P. Manns); The Beatson Institute for Cancer Research, Garscube Estate, Glasgow G61 1BD, UK (H. Y. Leung); Barbara Davis Center for Childhood Diabetes, University of Colorado Denver, Aurora, CO, USA (D. Maahs); Department of Experimental Medicine, University of Milano-Bicocca, Monza, Italy (F. Magni); European Medicines Agency, London, UK (E. Manolis); Medical University of Innsbruck, Department of Internal Medicine IV, 6020 Innsbruck, Austria (G. Mayer); Division of Nephrology, Department of Internal Medicine, University Medical Centre Groningen, University of Groningen, Groningen, The Netherlands (G. Navis); Department of Microbiology, University of Alabama at Birmingham, Birmingham, AL 35294, USA (J. Novak); Steno Diabetes Center, DK-2820 Gentofte, Denmark (F. Persson, P. Rossing); Baker Heart Research Institute, Melbourne, Vic. 8008, Australia (K. Peter); European Projects Office, Institute of Health Carlos III, Madrid, Spain (H. H. Riese); Department of Nephrology, Medical Faculty, University of Skopje, 1000 Skopje, Former Yugoslav Republic of Macedonia (G. Spasovski); Faculty of Medicine at Siriraj Hospital, Mahidol University, Bangkok 10700, Thailand (V. Thongboonkerd); Department of Nephrology, Ghent University Hospital, B 9000 Ghent, Belgium (R. Vanholder); Institut National de la Santé et de la Recherche Médicale (INSERM), U1048, Institut of Cardiovascular and Metabolic Disease and Université Toulouse III Paul-Sabatier,Toulouse, France (J. P. Schanstra); Division of Biotechnology, Biomedical Research Foundation, Academy of Athens, 11527 Athens, Greece (A. Vlahou).
